# Single-Docking Robotic-Assisted Radical Nephroureterectomy in Morbidly Obese Patient Post-Radical Cystectomy With Ileal Conduit: A Case Report

**DOI:** 10.7759/cureus.33466

**Published:** 2023-01-06

**Authors:** Bandar O Alsahn, Maher S Moazin, Raihanah S Al Khatem, Albaraa M Hariri, Fatimah A Alawami

**Affiliations:** 1 Urology, King Fahad Medical City, Riyadh, SAU; 2 Urology, Imam Abdulrahman Bin Faisal University, Dammam, SAU; 3 Urology, King Saud Medical City, Riyadh, SAU

**Keywords:** surgical management, transitional cell carcinoma, radical cystectomy, urothelial cancer, robotic nephroureterectomy

## Abstract

Bladder cancer is considered the most prevalent malignancy affecting the urinary tract system. Urothelial carcinoma, also known as transitional cell carcinoma (TCC), can arise from the entire urinary tract, with the bladder considered the primary site of origin and representing 95% of all cases. The management of TCC of the upper urinary tract is mainly by nephroureterectomy (NU). To our knowledge, there are no data regarding single-docking robotic-assisted NU following cystectomy with an ileal conduit. Therefore, in this study, we are reporting a case of single-docking robotic-assisted NU in a patient who previously underwent open cystectomy with an ileal conduit. A case of a 57-year-old female diagnosed with bladder cancer 10 years ago and underwent several transurethral resections of bladder tumor (TURBT) sessions presented for the first time in 2019, complaining of hematuria and dropping in the hemoglobin, which was not improving with multiple TURBT. For that, the patient underwent an open radical cystectomy with an ileal conduit. During the follow-up in 2021, computed tomography (CT) of the pelvis and abdomen with intravenous (IV) contrast showed a 7 mm enhancing lesion in the right proximal ureter, which was suspicious of proximal ureter mass. In 2022, the patient was again seen in the outpatient clinic; a CT of the pelvis and abdomen with IV contrast was done and demonstrated a significant progression of the mass size to 2 x 1.5 cm, with no other intraabdominal or intrathoracic lesions. For that, she underwent a single-docking robotic-assisted NU. To conclude, performing a single-docking robotic-assisted NU in a patient who previously underwent open radical cystectomy with an ileal conduit is challenging due to multiple adhesion and altered anatomy. More studies need to be published regarding the long-term outcomes of such procedures.

## Introduction

Bladder cancer is considered the most prevalent malignancy affecting the urinary tract system. Although multiple known risk factors contribute to the development of bladder cancer, cigarette smoking is the most prevalent and contributes to approximately half of all bladder cancer cases. Hematuria, microscopic or macroscopic, is the most common presentation of bladder cancer. In 75% of bladder cancer cases, urothelial bladder cancer (UBC) is diagnosed. Urothelial carcinoma, also known as transitional cell carcinoma (TCC), can arise from the entire urinary tract, with the bladder considered the primary site of origin and representing 95% of all cases. In comparison, upper tract urothelial cancer (UTUC) is uncommon and present only in 5 to 10% of cases [1,2].

Histopathologically, bladder urothelial carcinoma (BUC) and UTUC are similar; however, the latter possess a poorer prognosis and a higher risk of recurrence. The risk of having synchronous BUC at the time of UTUC diagnosis is 17%, while the opposite is much lower. For that, a patient diagnosed with UTUC has a higher chance of developing BUC [3].

The management of invasive, high-grade, or recurrent TCC of the upper urinary tract is mainly by nephroureterectomy (NU). In a recent study, the laparoscopic approach of NU possesses a significantly higher safety with lower hospital stay and activity limitations compared to the open technique. On the other hand, NU in a patient who underwent radical cystectomy with an ileal conduit is considered a complex procedure and difficult to approach due to the multiple adhesions and altered anatomy [4].

To our knowledge, there are no data regarding single-docking robotic-assisted radical NU following cystectomy in a patient with an ileal conduit. Therefore, in this study, we are reporting a case of single-docking robotic-assisted NU in a patient who previously underwent open cystectomy with an ileal conduit.

## Case presentation

**Figure 1:**
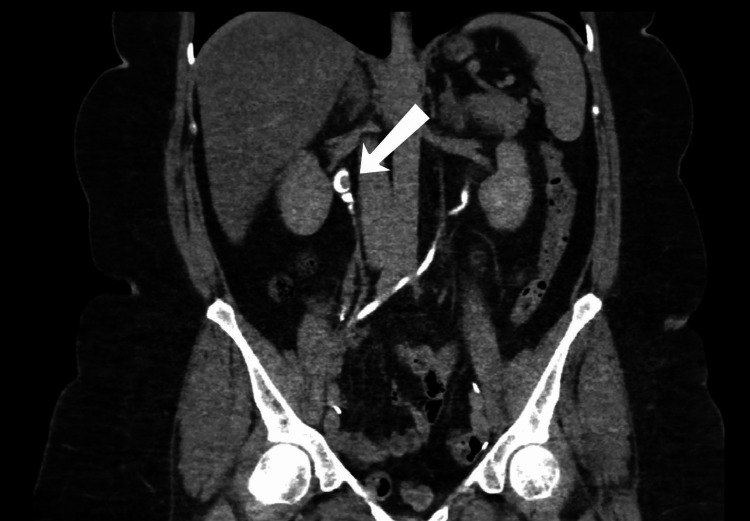
Pelvis and abdomen computed tomography with IV contrast showed a 7 mm enhancing lesion in the right proximal ureter. IV: intravenous

**Figure 2:**
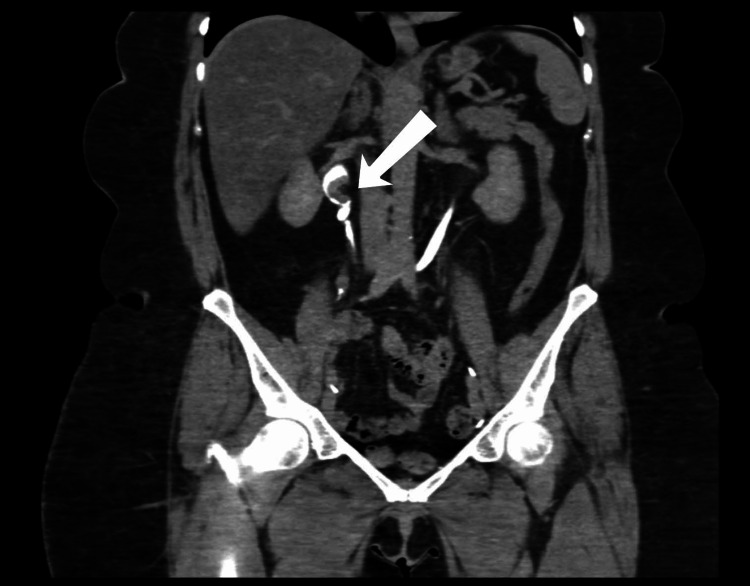
Repeated pelvis and abdomen CT with IV contrast showed a significant mass measured 2 x 1.5 cm. CT: computed tomography; IV: intravenous

A 57-year-old morbidly obese female with a body mass index (BMI) of 39.56 kg/m^2^. She is a known case of bladder cancer and underwent multiple transurethral resections of bladder tumor (TURBT) over the previous 10 years. The patient’s first presentation to the hospital was in 2019, as she complained of hematuria and dropping in the hemoglobin, which was not improving with multiple TURBT for which an open radical cystectomy with an ileal conduit was done. The histopathological report showed a tumor size of 8.5 cm, a high-grade lesion, with invasion to the muscularis propria, and staged as p2T N0 M0. During the follow-up in 2021, computed tomography (CT) of the pelvis and abdomen with intravenous (IV) contrast was done. It showed a 7 mm enhancing lesion in the right proximal ureter, which was suspicious for a proximal ureter mass with no hydronephrosis (Figure 1). For that, the case was discussed by a multi-disciplinary team, and a decision was made to go for a radical NU. The patient was consulted for a robotic right radical NU; however, the patient lost follow-up. In 2022, the patient was again seen in the outpatient clinic; a CT of the pelvis and abdomen with IV contrast was done and demonstrated a significant progression of the mass size to 2 x 1.5 cm, with no other intraabdominal lesions (Figure 2). Moreover, a CT scan of the chest showed no evidence of intrathoracic metastasis. For that, the patient was scheduled for a single-docking robotic-assisted radical NU.

**Figure 3:**
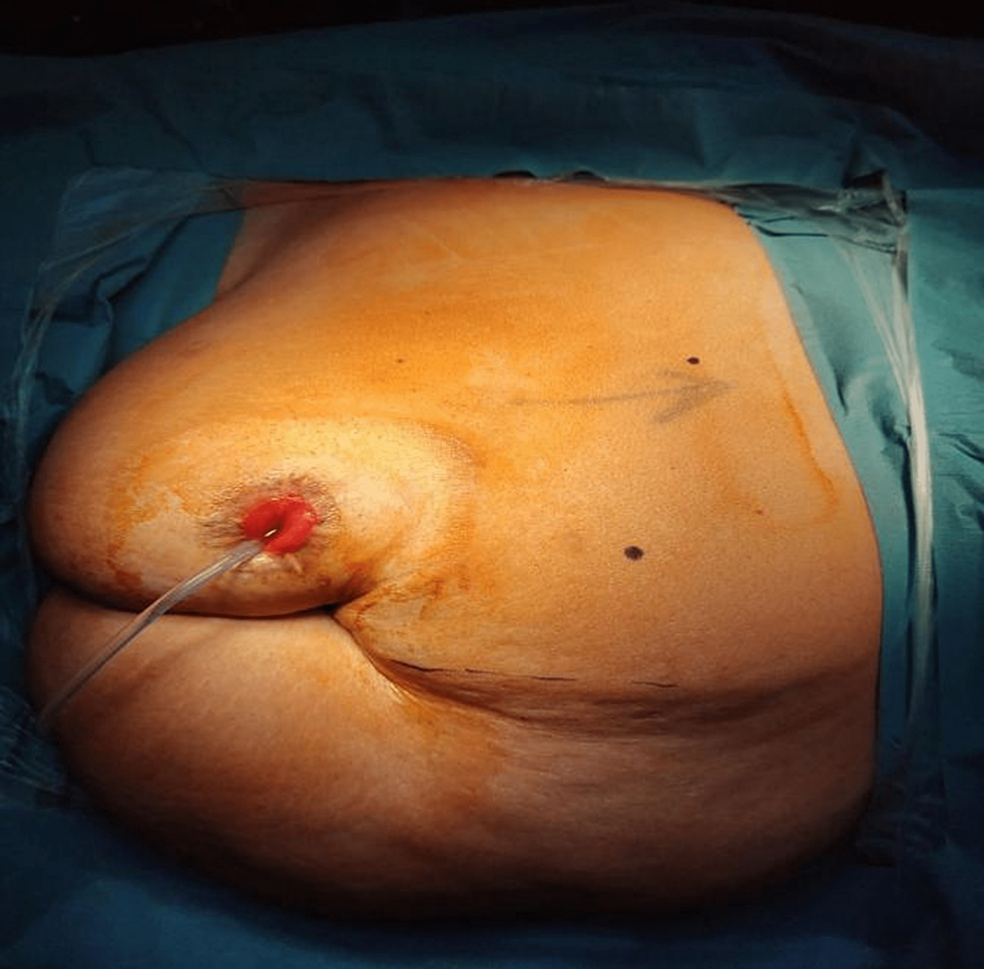
Patient at left lateral decubitus position with previous ileal conduit.

**Figure 4:**
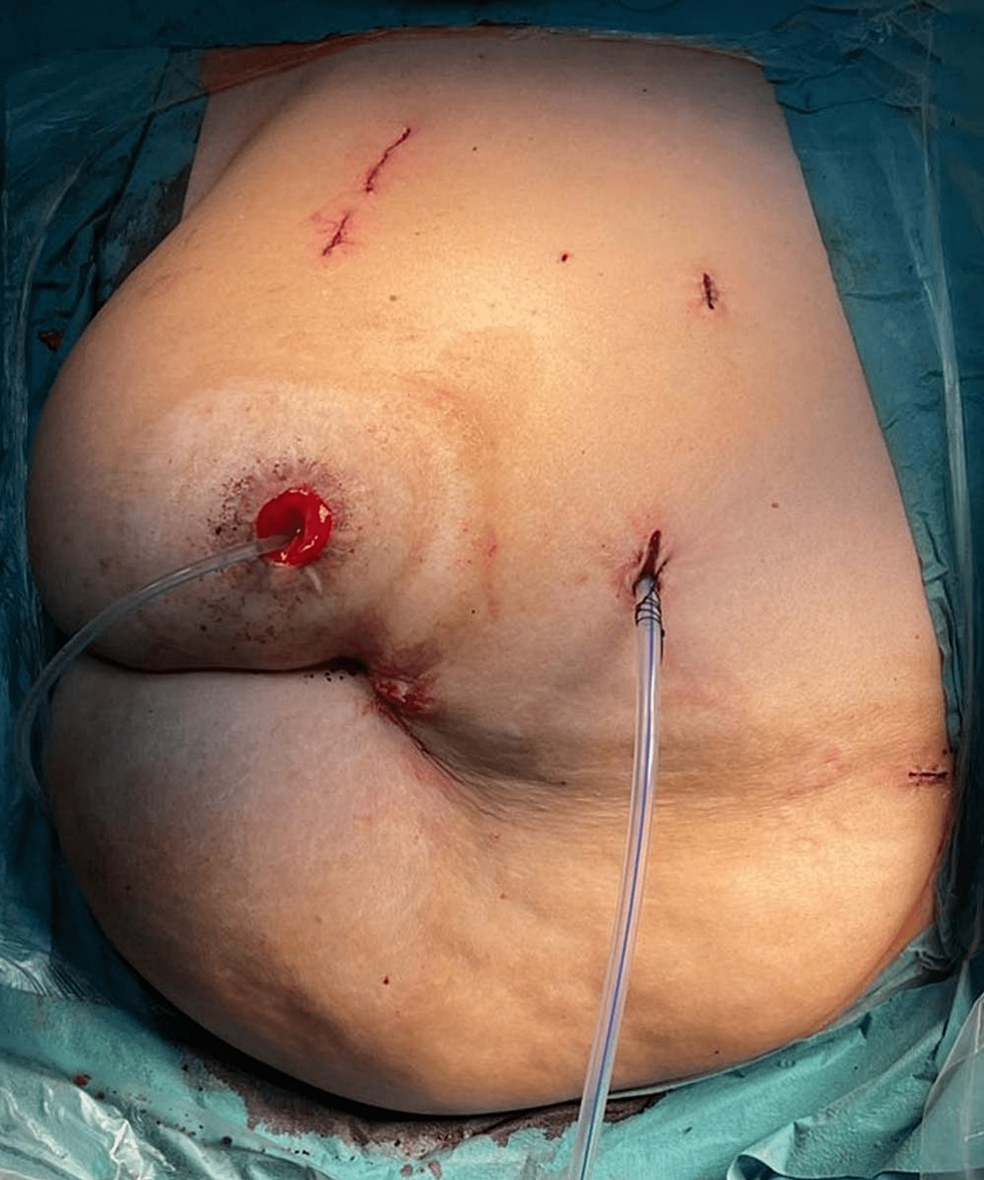
Surgical drain and wounds postoperatively.

**Figure 5:**
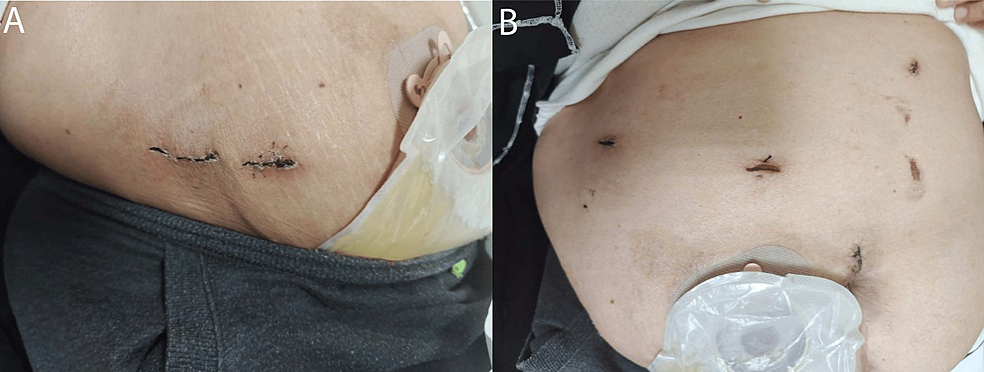
Proper wound healing with no signs of infection two weeks postoperatively.

During the procedure, the patient was placed and secured in the left lateral decubitus position at a 45° angle. The lower limb flexed at 90° and the upper limb extended with adequate cushioning (Figure 3). The camera trocar was placed along the right lateral edge of the rectus sheath, slightly cephalic to the umbilicus, and the 30° lens was inserted under direct visualization. After examination of the abdominal cavity, the other robotic trocars were placed along the lateral edge of the rectus sheath. An assistant port was placed in the midline above the umbilicus, and another port was inserted to retract the liver (Figure 4). The total operative time was six hours, and the estimated blood loss was approximately 100 mL. The patient was shifted to the surgical ward postoperatively, and she tolerated the procedure well. The postoperative course was uneventful, and the patient was discharged home on the third day postoperatively. There were no significant changes in the hemoglobin postoperatively (14.7 vs. 14.4 g/dL). In addition, creatinine was elevated (124 vs. 88 umol/l). The histopathological report showed non-invasive high-grade papillary urothelial carcinoma of the proximal ureter with negative margins and no lymphovascular invasion. The patient was followed up two weeks after the operation and showed proper wound healing with no signs of infection (Figure 5).

## Discussion

Reviewing the literature shows no published cases concerning single-docking robotic-assisted NU approach following an open cystectomy with an ileal conduit in a morbidly obese patient.

In 2006, Romero et al. published a case series reporting their experience in laparoscopic NU following radical cystectomy with urinary diversion as management for upper urinary TCC. They included seven patients who underwent this procedure, and the average operative time was 305 minutes, and most of the time was consumed in excising the ureter from the urinary diversion. Moreover, estimated blood loss among patients ranged from 100 to 250 mL; however, they did not mention the change in hemoglobin level before and after the procedure. In addition, the hospital stay was approximately 11 days, with a minimum of five days [4].

In 2008, Barros et al. released a review of eight patients who underwent simultaneous laparoscopic NU and radical cystectomy for bladder cancer with synchronous upper urinary tract tumor. The mean estimated blood loss was approximately 755 mL, the operative time was nine hours, and the length of hospital stay was 7.5 days [5].

As we compared the previous cases to this case, our patient had a lower estimated blood loss of approximately 100 mL and no significant drop in hemoglobin. Also, the patient had a shorter operative time of six hours, and a shorter length of hospital stay as the patient was discharged home on the third postoperative day.

## Conclusions

UTUC is an uncommon type of cancer that present only in 5 to 10% of all cases. However, UTUC had a poorer prognosis and a higher risk of recurrence than UBC. For that, it is managed mainly by NU. The robotic-assisted NU approach is considered a new modality with better efficacy and safety in various conditions. Moreover, performing a single-docking robotic-assisted NU in patients who previously underwent open radical cystectomy with an ileal conduit is challenging due to multiple adhesion and altered anatomy. Therefore, further studies should be conducted regarding this procedure to evaluate its efficacy, safety, and long-term outcomes, as it can be a more feasible approach in such patients.
